# Shark Fin Consumption May Expose People to Neurotoxic BMAA

**DOI:** 10.1289/ehp.120-a191

**Published:** 2012-05-01

**Authors:** Wendee Holtcamp

**Affiliations:** Award-winning freelance writer Wendee Holtcamp has previously written about sharks and scuba dove with them while reporting for Discovery Channel’s *Shark Week*.

Shark-fin soup has gotten a bad rap since conservation groups began raising awareness of shark finning, a practice in which fishermen slice the fins off the animals, sometimes while they are still alive, and discard the bodies overboard. Now, scientists have found another reason to avoid the Asian delicacy: it may be detrimental to neurologic health.

Worldwide, an estimated 26–73 million sharks are traded on the fin market annually.^1^ Shark fin and cartilage are also sold as powder or in capsules, and traditional Chinese medicine claims it nourishes the blood, enhances appetite, and energizes multiple internal organs.^2^ However, the U.S. Food and Drug Administration has not confirmed any health benefits, and a 2004 court injunction required one company to pay restitution to customers who purchased shark-fin products falsely claiming to treat cancer and HIV.^3^

Deborah Mash, a neuroscientist at the University of Miami Medical School, led the first study to show shark tissues contain the neurotoxic amino acid β-methylamino-l-alanine (BMAA).^4^ The nonprotein amino acid is produced by cyanobacteria (sometimes called “blue-green algae”), which are often associated with nutrient runoff in coastal waters, although huge blooms also occur in the open ocean. BMAA has received attention due to increasing evidence that consumption of contaminated food or water may contribute to amyotrophic lateral sclerosis, Alzheimer disease, and Parkinson disease.^5^

Mash collaborated with Neil Hammerschlag, a University of Miami research assistant professor, who captured sharks in Florida coastal waters. BMAA was detected in 23 of 29 fin samples and in all species tested—nurse, great hammerhead, lemon, bull, bonnethead, blacknose, and blacktip sharks—at concentrations of 144–1,836 ng/mg wet weight. BMAA levels in shark fins fell within the range Mash measured in a previous study^6^ of the brains of humans who died from neurodegenerative disease, which she says suggests BMAA has biomagnified in these top predators.

The scientists also tested a few samples of organ tissue from great hammerheads killed in recreational fisheries. BMAA levels averaged 1,450 ng/mg in kidney, 588 ng/mg in liver, and 58 ng/mg in muscle. None was detected in heart tissue. By comparison, eight hammerhead fins averaged 1,028 ng/mg.

**Figure:**
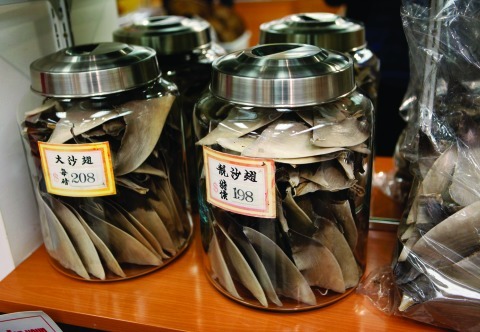
Shark finning is a lucrative practice—these shark fins are being sold for $208 and $198 a pound at this store in San Francisco’s Chinatown in 2011. In Chinese culture, serving shark-fin soup is a symbol of wealth and good fortune. © AP Photo/Paul Sakuma

“What the shark is teaching us is that, like man, apex predators are probably getting exposed to BMAA through their diet,” says Mash. Past studies have detected high levels of BMAA in some fish and crustaceans in Florida waters, including pink shrimp and blue crabs, which both humans and sharks consume.^7^

Sharks typically contain high mercury levels,^8^ and a recent study by Doug Lobner, a biomedical science professor at Marquette University, showed that mercury and BMAA act synergistically to kill neurons at levels lower than either substance alone.^9^ Multiple environmental neurotoxicants combined with a genetic risk factor “could be precisely the one–two punch to put you at increased risk for a degenerative brain disease,” Mash speculates.

The Florida shark population sampled likely makes up a relatively small portion of the global fin trade, but the findings have implications for sharks worldwide. “The shark [in Florida] is biologically the same as anywhere else, and there’s cyanobacteria everywhere,” Hammerschlag says.

Shelley Clarke, a shark-fin trade expert at Imperial College, London, says it is safe to assume that all the species tested in the paper are used in the shark-fin trade. “Some of the species have been confirmed present in the trade through genetic analysis,” she says.

Great hammerhead fins are highly valued, and blacktip and bull sharks have extensive global distributions, according to Mahmood Shivji, director of the Guy Harvey Research Institute at Nova Southeastern University in Fort Lauderdale, Florida. “Certainly if BMAA occurs in great hammerhead fins that come from parts of the world where they are captured for the fin trade, that is a likely avenue for human consumption,” he says. BMAA does not change when it’s cooked,^10^ but Clarke says anecdotal evidence from the Hong Kong fin trade suggests some fins may be treated with formaldehyde, and it is not certain whether such a practice would change BMAA bioavailability.

Still, more research is needed to make a case for human risk. “To make a stronger case, it would be useful to look at fins obtained from the market and from species most common in the fin trade,” says Shivji (those include blue sharks,^1^ which were not tested because they are not found in the study area). “Consumption risk assessments done for pollutants in food require estimation of concentrations and calculations of consumption in relation to consumer body mass and development stage.” However, the results suggest that consumption of shark-fin products may pose a significant health risk for degenerative brain diseases.
